# The struggle over caesarean section on maternal request: an ethical principles approach to Swedish media portrayal

**DOI:** 10.1186/s12978-025-02057-3

**Published:** 2025-06-27

**Authors:** Mio Fredriksson, Magdalena Mattebo

**Affiliations:** 1 Department of Public Health and Caring Sciences, Health Services Research, Uppsala University, Box 564, Uppsala, 751 22 Sweden; 2 School of Health, Care and Social Welfare, Division of Caring Sciences and Health Care Pedagogics, Mälardalen University, Box 883, Västerås, 721 23 Sweden

**Keywords:** Caesarean section on maternal request, Cesarean birth on maternal request, Ethical principles approach, Sweden

## Abstract

**Background:**

Caesarean section on maternal request (CSMR) is a complex ethical issue. Sweden is an intriguing case for studying CSMR due to its restrictive policy that curtail women autonomous choice regarding the mode of birth, despite overall policy developments aiming to strengthen patients’ rights, promote person-centred care, and adopt a more individualized approach. This study aims to understand how the tension surrounding CSMR is manifested in Swedish media. An ethical principles approach is used to investigate what image of CSMR is created and conveyed to the public and what main arguments are used for and against CSMR.

**Methods:**

This study covers news media and specialist press material in Sweden between March 2019 and March 2024. Searches were carried out in Retriever Research and resulted in 188 search hits. The content analysis had both an inductive and a deductive approach based on Beauchamp and Childress’s four ethical principles: respect for autonomy, non-maleficence, beneficence, and justice.

**Results:**

The analysis reveals a struggle in Swedish media over how to approach CSMR, touching on all ethical principles. Regarding *autonomy*, the debate focuses on whether CSMR is a medical decision about non-indicated surgery or a private choice. In terms of *non-maleficence*—which is the health professionals’ main concern—the media highlights conflicts over interpreting risks associated with caesarean section and vaginal birth. *Beneficence* involves a tension between portraying vaginal birth as healthy and normal versus caesarean section as a method to protect sexual and bodily function. As regards *justice*, CSMR is portrayed as a challenge to the fundamental needs-principle in the healthcare system.

**Conclusions:**

Ultimately, the ethical principles approach to analyzing the media portrayal of CSMR in Sweden highlights the need for decision guidelines and practices that are more open to a woman’s own evaluation of different risks while also encouraging critical reflection on the underlying factors shaping treatment preferences for both women and healthcare professionals.

**Supplementary Information:**

The online version contains supplementary material available at 10.1186/s12978-025-02057-3.

## Introduction

Childbirth is a cultural, religious and political phenomenon, beyond being a biological and personal experience [1, 2]. A notable worldwide trend is the increasing rate of caesarean section (CS) [3–6], which is now one of the most common surgical procedures [7]. In many countries, the rate of CS goes far beyond the 10–15% recommended by the WHO (Supplementary material 1), up to which maternal and neonatal mortality decreases [8, 9]. Drivers for the increased rate of CS are medical and obstetric factors such as an increase in maternal age and obesity, and multiple pregnancies and assisted reproductive technologies. However, one of the drivers is also CS on maternal request (CSMR), also referred to as cesarean birth on maternal request, patient choice cesarean or elective cesarean section [10, 11]. It is a procedure that implies carrying out a CS in the absence of medical indications for avoiding vaginal birth, such as abnormal fetal presentations, placental pathology, etcetera [7]. It is to some degree controversial and associated with many ethical questions [12]. According to McFarlain [13], CSMR has become attractive to both physicians and women among other things because of the safety of the procedure, liability issues associated with vaginal birth, an increasing awareness of the prevalence of urinary and fecal incontinence after childbirth, and the cultural importance of autonomy and the right to choose. Women’s reasons for wanting an elective CS include fear of labor pain, anxiety for fetal injury/death, fear of childbirth, urinary incontinence, pelvic floor and vaginal trauma, experience of prior bad birth, but also doctor’s suggestion and planning reasons [3, 14].

Accurate comparative statistics on CSMR is lacking [12, 15], but it is clear that there is a great variation between countries. Begum et al. suggest that factors associated with a higher share of vaginal births may be strict policies on CSMR, the legal framework for medical litigation, cultural or social pressure and midwifery-led approaches to births [5]. In contrast, a higher degree of private funding in the health system increases the rate of CSMR [16, 17]. Undoubtedly, fear of legal consequences or malpractice claims is a driver for many obstetricians to perform CS [7]. However, this is not the case in Sweden [18], which is the empirical focus in this article, where physicians report a low level of fear of legal consequences or being blamed in cases of adverse outcomes [19]. As in other Nordic countries, CS rates are low in Sweden: 17.4% compared to 27.2% in what Begum et al. refer to as more developed countries [4]. It is estimated that 1–2% of primiparous deliveries are CSMR and 3–7% of multiparous deliveries [20]. In contrast to some countries where the discussion is about women being pressured by health professionals into CS—primarily for physician convenience and to avoid malpractice claims [21, 22]—much of the debate in Sweden centers around women being denied elective CS, despite their preference, leading to considerable dissatisfaction among pregnant women [23]. Another country, also with a tax-based healthcare system, where it has been noticed that women are being denied elective CS is the UK [24].

Sweden is an intriguing case for studying CSMR because it highlights challenges in meeting individual preferences in a collective and needs-based healthcare system. On the one hand, current guidance on CSMR is restrictive and curtail women’s autonomous choice regarding the mode of birth [25]. On the other hand, there is an overall policy drive to strengthen patients’ rights, and recent national policies promote person-centred care, a more individualised approach, and increased patient choice [26]. This situation creates uncertainty for both pregnant women and health professionals, and CSMR is currently being discussed at the clinical level, by knowledge-producing authorities, and is also debated in the media. Research has shown that the media appear to influence how women engage with childbirth, as portrayals of normal birth are often missing and birth is portrayed as risky, dramatic and painful [27, 28]. For most people, media portrayals shape their perception of CSMR, as few may have first-hand experience, highlighting the importance of exploring this topic. This article aims to understand how the tension surrounding CSMR is manifested in Swedish media. In a qualitative content analysis, an ethical principles approach is used to investigate what image of CSMR is created and conveyed to the public and what main arguments are used for and against CSMR.

Before describing the methods, the previous literature addressing CSMR as an ethical issue is summarized, and the most relevant aspects of the Swedish context are outlined.

### Caesarean section on maternal request: an ethical challenge

Some scholars have addressed this ethical challenge using Beauchamp and Childress’s [29] four ethical principles, however, arriving at different conclusions regarding recommended action. The principles include respect for *autonomy* (compliance with self-determination), *non-maleficence* (do no harm), *beneficence* (maximizing benefit), and *justice* (equity and non-discrimination). Sometimes beneficence is considered to encompass non-maleficence [12] and they may overlap.

There are different standpoints regarding CSMR based on whether autonomy is specified in terms of negative or positive rights: “that is, the right to refuse a certain intervention, or the right to demand it” ([12], p. 5). The demand for an intervention is less supported than refusing it is [12]. Romanis argues that the individual’s right to bodily integrity is crucial and that this right should not be diminished by pregnancy [24]. A key question is whether negative rights apply to vaginal birth or not. For example, it has been argued that women know that vaginal birth “is the normal and inevitable physiological consequence of carrying a pregnancy”, that “it is a fact of pregnancy” ([24], p. 263). In this interpretation, bodily integrity is not diminished by denying a woman a CSMR.

Critics of CSMR argue that women should not be allowed to exposing themselves (or the fetus) to potential additional harms or risks [24], such risks being increased risk of maternal mortality, risk of infection or hemorrhage, risks from anesthetics or respiratory distress in neonates. In this way, the principle of non-maleficence can justify an interference with autonomous choice. When considering invasive procedures, for most health professionals non-maleficence outweighs patient autonomy [13]. However, vaginal deliveries may also result in adverse outcomes for the woman, such as damage to the pelvic floor and urinary incontinence [12]. There is thus no guarantee that vaginal deliveries are non-harmful [24], although there is a difference between adverse outcomes resulting from a natural bodily process and those resulting from a medical intervention. As argued by Romanis [24], there may be rational reasons to why a woman may prefer some risks over others or assumes some types of risks to get reassurances; individuals have different perceptions of risk, so have pregnant women. Romanis further argues that women should be “entitled to decide what physical consequences of reproduction they are willing to assume” ([18], p. 251).

At the core of the CSMR-question is that the procedure is performed in the absence of clinical need. What clinical need is, however, is largely decided by health professionals, and is framed in terms of medical need. Thus, it has been argued that it would be different with a more holistic approach to physical and mental health [24, 30], for example taking into consideration negative experiences from childbirth or worries about sexual function and relationships [24]. It has also been argued that the principle of beneficence should take into consideration non-medical interests the pregnant woman may have. Although many studies indicate, for example, that the frequencies of maternal mortality and maternal morbidity are higher after CS than after vaginal birth, and that CS is associated with increased risks of uterine rupture, abnormal placentation, ectopic pregnancy [31], there is not an agreed evidence base [12]. Many studies of CS risks use data from emergency CS or from procedures where there were indications for CS, making effects more inconclusive [13, 24]. However, comparing elective non labour cesarean birth to planned vaginal birth, benefits and burdens appear equal [12]. In practice, the risks of CS are not so often compared to the risks of vaginal births, that are not risk free either [24]. Brown and Mulligan [30] (among others) stress that injuries from vaginal births are ”routinely underestimated in discussions about childbirth”, perhaps because vaginal birth is seen as inevitable and injuries therefore regarded unavoidable. It could therefore be argued that denying CSMR may cause significant harm [24].

Regarding justice, the argument is that costlier treatments or interventions should not be used if benefits are not tangible, and putting additional resources in one area may have adverse impact on other areas [12]. Some have argued that CSMR should not be publicly funded for reasons of justice, or patients pay the additional cost, preventing “their choice becoming an unfair burden on others” [24]. It has been a common proposition that costs for CS are higher compared to vaginal birth (additional costs are related to preoperative checks, and the procedure (including e.g. anesthetic, surgical time and post-operative hospital stays [24]), although some studies have concluded that differences may not be substantial [32, 33], in particular if compared to medically supervised vaginal births. Costs from long-term effects of different modes of birth are also difficult to control for, even though for example surgery for adhesions in the intestine and ileus are pointed out as long term consequences [20].

### Caesarean section on maternal request in Sweden

The Swedish healthcare system is of collective nature. It is funded through taxes and offers relatively few individual patient rights due to its redistributive nature, which is based on medical need and not demand [34]. The 21 regions are according to law (SFS 2017:30) obliged to provide good healthcare to their inhabitants. However, this does not correspond to an individual patient right to get certain treatments, and the actual care provided to patients depends on for example local resources, staffing, and priorities (termed quasi-legal rights [35]). However, there is an overall wish to strengthen patients’ rights, and recent national policies promote person-centred care, a more individualised approach, and increased patient choice [26]. The law stipulates that the patient’s autonomy and integrity are to be respected (SFS 2017:30 Chap. 4, 1§) and that healthcare shall, as far as possible, be formulated and carried out in consultation with the patient (SFS 2017:30 Chap. 5, 1§).

Current national guidance issued by a group of Swedish authorities and The Swedish Society of Medicine states that a woman must have a *sufficiently compelling reason* to give birth via CSMR, which is to some extent open to health professionals’ individual interpretation [25]. It specifies that a high degree of fear of birth can be a factor, as can acute psychiatric illness and being a victim of sexual abuse; but not age in itself, not previous CS in itself, not previous birth injuries such as significant tearing and incontinence (unless there are serious remaining problems), usually not a previous stillborn or injured infant related to pregnancy or birth, and not practical reasons such as planning. It is concluded that a woman’s request for a CS should be accommodated if her reasons are regarded sufficiently compelling and she maintains her request after receiving relevant information and being offered supportive discussions or other forms of care [25]. Fear of birth is handled at specialized units, often called *Aurora clinics*, which provide individualized support. These are available at all maternity units in Sweden, though their organization, resources, and other factors may vary. The goal of these specialized units is typically to boost the pregnant woman’s confidence before birth, reduce fear and anxiety, and promote a positive childbirth experience regardless of the birth method. The support is tailored to the individual’s needs and may include information about the childbirth process, strategies for managing labor pain, and relaxation and breathing techniques [36].

Traditionally, there is a culture of belief in ‘normal birth’ in Swedish maternity care services, meaning it offers best possible outcomes for women and infants [19, 37]. This is visible in a restrictive approach to CSMR [25]. Recent studies show that while Swedish health professionals aim for shared decision-making regarding the mode of birth, they ultimately see it as a professional decision, prioritizing the protection of women’s long-term health over responding to current preferences [38]. Furthermore, a recent report also shows that pregnant women and health professionals have differing views on whether women should have the right to choose CS [20]. This is also the claim made by advocacy groups being active on social media and in traditional media. Most visible is the group Rätten att välja kejsarsnitt (The right to choose caesarean section, RVK) with about 7,500 members.

## Methods

### Study design

Qualitative content analysis was used to investigate what image of CSMR is created and conveyed to the public and what main arguments are used for and against CSMR. The study covered news media and specialist press material in Sweden between March 2019 and March 2024, that is a five-year-period. Searches were carried out in Retriever Research, which is the largest digital media archive in the Nordic countries and collects news from print and digital editorial media (as well as radio and television). All print and web sources were included (4,264 sources). The search covered four search terms in Swedish, Table 1. These search terms were chosen after searching different time periods and media sources to investigate which terms were used in the reporting about CSMR.

**Table 1:** Search terms and results

English term	Swedish term	Search result
Right to caesarean section	Rätten till kejsarsnitt	15
Caesarean section on maternal request	Kejsarsnitt på moderns önskan	18
Choose caesarean section	Välja kejsarsnitt	143
Caesarean section without medical reasons	Kejsarsnitt utan medicinska skäl	12

The search resulted in 188 search hits, of which some were news articles or news items published by several sources. Most search hits were noted during 2019 and 2023 (46 and 45, respectively), while there were fewer between 2020-2022 (31, 20 and 26, respectively) and ten during the first three months of 2024. The search term “choose caesarean section” yielded the most results. Three sources published most on CSMR, Dagens Nyheter, Göteborgs-Posten and Aftonbladet (24, 16 and 13 articles, respectively). However, the topic was covered by both larger and smaller news media throughout Sweden.

### Data analysis

The study had both an inductive and a deductive approach. To begin with, all media material found in the database search was read, inductively coded with a focus on the presented arguments, and sorted into themes (the 188 search hits). The coding was performed by MF. After that, a deductive content analysis [39] based on Beauchamp and Childress’s [29] four ethical principles was employed to analyze the media material, triangulated by MM. This meant that the themes were sorted under the four principles and the main arguments distilled, presented in Table 2. Some arguments may have bearing on more than one principle, as the principles are interlinked. The analysis is based on the 188 search hits. Additional file 2 presents the media sources cited in the results section, which illustrate the full material.

**Table 2:** Summary of arguments for and against CSMR

Autonomy	Non-maleficence	Beneficence	Justice
*For CSMR*	*For CSMR*	*For CSMR*	*For CSMR*
• Women should have the final decision on how to give birth, it is not *any* type of task • It is not a medical decision, but a private one • It is the woman who has to live with the consequences, not the obstetricians	• It is abuse to force someone to do something they do not want to do (vaginal birth) • Forced vaginal birth may lead to trauma • The psychological risks are poorly researched • Risks from vaginal birth are under reported: it is not risk free either • The risk calculations are misleading: acute and planned CS are confused • It may be a strategy for self-preservation	• Avoiding fear of birth before and during birth • Optimizing the start of parenthood • Protecting sexual and bodily function	
*Against CSMR*	*Against CSMR*	*Against CSMR*	*Against CSMR*
• It is the woman’s right to her own body driven into absurdity • Women must accept that a pregnancy ends with a vaginal birth (which can be painful) • Women and health professionals are not equal when it comes to knowledge • Women should not have the right to make decisions about major abdominal surgery	• All procedures should have as low medical risk as possible: risks are higher with CS	• Vaginal birth is natural, normal and healthy: the best medical approach • CS does not operate away mental ill health • Women with phobia should get evidence-based treatment • Women should get supported to give birth vaginally and safely, • Birth contracts can be offered instead	• From a societal perspective, it is not desirable with a larger share of CS • Assessments are arbitrary • There may be crowding-out effects/go against the needs-principzle

### Ethical concerns

The Swedish Ethical Review Authority did not have any objections to the study (2024-00637-01). However, because we are interested in the arguments used for and against CSMR rather than who is making the arguments, no names are mentioned in the results presentation.

## Results

### A well debated phenomenon: CSMR being a polarized issue

The inductive reading of all search hits demonstrated that CSMR is portrayed in different types of media articles. There are news articles and feature articles as well as opinion pieces by editorial writers and opinion pieces by advocates for CSMR and members of the public. The final sources are presented in Supplementary material 2. Important stakeholders interviewed in news and feature articles were obstetricians, midwives, pregnant/previously pregnant women as well a national-level experts and representatives of advocacy groups (primarily the RVK-group, The right to choose caesarean section). Potential stakeholders that were almost unmentioned were the foetus and the pregnant woman’s partner.

Overall, CSMR is constructed as a contentious and polarized issue with hard opinions on what is right or wrong, and with furious debates. It is framed as a confrontation between, in some cases physicians and women and in some cases midwives and women, with physicians with a more permissive attitude in between. For example, in a debate article signed by 300 women it read: “*It sails up to battle over the question of who has the right to decide how a woman should give birth. Is it a medical issue for doctors or should the woman be allowed to decide over her own body?*” ^[S1]^ Some go as far as saying that it a form of obstetric violence women are subjected to when they want an elective CS but are denied one ^[S2]^.

The increasing wish for a CSMR is by some discussed from the perspective of modernity and the change over time in what is perceived as a successful birth, embedding a constant strife towards something more modern, and distancing oneself from “what has been” ^[S3]^. It is argued that pregnancy and childbirth are permeated by changing ideas and rules that are moral, ideological as well as medical ^[S4]^. At the same time, pregnancy and vaginal birth are framed as “natural” and “healthy” ^[S5]^. One woman describes how she perceived childbirth was thought of by people around her: “*A vaginal birth shows that the woman is strong and can take the unbearable pain*,* and that her partner can be proud to have such a woman*,* a real ‘warrior’*” ^[S6]^. In this view, CSMR is undesirable. CSMR is furthermore linked to consumerism, that the customer is always right and that the professionals should just obey ^[S7]^. It is also described in terms of a previous “life knowledge” ^[S8]^ that is about to get lost, which means that people before were able to adapt to a situation they could not control or plan for. At the same time as the increase in women wanting an elective CS is linked to an enhanced consideration of “body and soul” ^[S8]^ from the part of the health services, midwives are accused of being against medical advances, being backward-looking and seeing CSMR as an overmedicalization ^[S9]^.

### Autonomy: both understanding and dismissal

The main argument put forward by the RVK-group is that in an equal society, women should have the final decision on how to give birth ^[S10]^. They argue that it is not *any type* of decision or task, but concerns how to bring new lives into the world. They argue that it is not a medical decision and that women must be able to decide on this “private matter” ^[S11]^, also that it is not the obstetrician who has to live with the consequences, but the woman who may have to face life with trauma and injuries ^[S12]^. A woman in favor of autonomy says: “*It is our bodies it is about*” ^[S6]^. An analogy is made to abortion, where women are free to decide without anyone asking about the reason ^[S11]^. Members of the RVK-group also argue that women can make well-informed decisions but that the maternity care services declare them incompetent by denying them this opportunity: “it’s downright offensive” ^[S13]^. This is contested by some debaters, arguing for instance that CSMR is “the woman’s right to her own body driven into absurdity” ^[S7]^ and the same thing as people with a cold demanding “to be sedated in the emergency room” ^[S14]^, i.e. “pure madness” ^[S7]^. These debaters also present such arguments as: “*If you still do not think that the pain of childbirth is in proportion to the joy of having a child*,* you should perhaps consider whether a dog is not a better alternative*” ^[S14]^. They argue that one must accept the premises that a pregnancy ends with a vaginal birth, unless there are medical reasons for a CS.

Some debaters claim that CSMR de-professionalizes the physicians and midwives, which have both knowledge and experience, and should therefore be the ones making the decisions ^[S7, S14]^. They resist the attitude that women and physicians are equals when it comes to knowledge—"it is a delusion" ^[S14]^— and argue, for example, that googling cannot be compared to medical training, that it is easy to confuse information—which pregnant woman can get—with knowledge. Some physicians also reason in a similar manner, expressing for example that it is “*out of the question*” that women should have the right to make the decision about CSMR as it is a major abdominal surgery (not childbirth), and could be dangerous if not indicated by medical necessity ^[S1]^. In one of the media articles, a woman expressed her experience with a physician with this type of attitude: *“The physician I met was cold and unsympathetic. She showed with all clarity that she had all the power*,* that I was completely in her power*,* and that she was not going to help me”*^[S2]^. However, other physicians express a more balanced approach, for instance that they understand the wish for autonomy: “*It is the woman who is exposed to the risk and should therefore be able to decide for herself*” ^[S15]^. They however, also talk about professional ethics and responsibility, and maintain that it must be a combination and compromise of different perspectives. In the end, although decisions should be made jointly, it is ultimately the obstetrician who must make the final decision based on each woman’s individual circumstances.

### Non-maleficence: what is evidence?

In the investigated media material, a health professional explains that the healthcare services proceed from the perspective that all interventions should be as safe as possible, i.e. have as low medical risks as possible, which is why CSMR is not the preferred option, but vaginal birth ^[S16]^. It is a clear expression of the do no harm-principle. Evidence regarding the risks of CS and vaginal birth forms the core of the argumentation as regards the approach to CSMR, but there is a clear struggle to define what is regarded as evidence. In many of the media articles, there are accompanying information boxes describing current evidence for and against vaginal birth and CS ^[S16, S17]^. The complications that can occur from CS are described as more serious than those from vaginal birth. It is pointed out that childbirth is safe in Sweden, with few complications, but that the risk for serious complications is higher with CS ^[S10]^. Several physicians express that they do not operate unless it is necessary, and it is summarized that “its ingrained to not allow unnecessary CS” ^[S13]^ and “to keep the CS rate down” ^[S18]^.

However, in the media, there is a contestation of the evidence presented by physicians, midwifes and the health services. Firstly, there is a critique against what types of risks are presented and compared. For example, members of the RTK-group argue that they understand that CS is not risk free, but maintain that neither are vaginal births, and that injuries from vaginal births are under-reported: vaginal births thus being easier to “sing praise to” ^[S19]^. One woman explains that she preferred the risks associated with a CS over those associated with vaginal birth, but was denied elective CS. “*I was not afraid of giving birth*,* I preferred the risks with CS to those with vaginal birth*” ^[S2]^. Other women testified to the low attention to injuries associated with vaginal birth: “*I was persuaded and brainwashed by the healthcare services that vaginal birth was the best. They concealed all the problems with vaginal birth and talked about how fantastic the postpartum care was and that injuries were rare. Instead*,* they described how terrible a C-section was. After that*,* I felt forced and eventually agreed to a vaginal birth.*” ^[S20]^ The RVK-group also argues that risks with CS presented by the Aurora clinics and other healthcare providers, confuse planned and acute CS. Risks and costs from *acute* CS are compared to vaginal births instead of risks and costs with *planned* CS, which is misleading for decisions about CSMR ^[S2]^.

Secondly, there is a critique against the lack of evidence relating to ‘psychological risks’ ^[S18, S20, S21]^. It is argued that the psychological consequences of not being able to give birth in the preferred way are poorly researched, but the RVK-group refers to research that has shown that women who have been denied CS and instead given birth vaginally more often suffer from PTSD and depression compered to women who have given birth in the way they preferred ^[S12]^. In addition, members of the RVK-group dispute how effective Aurora care really is, based on a recent report from the Swedish Agency for Health Technology Assessment and Assessment of Social Services establishing that there is no scientific evidence for the Aurora care ^[S21]^.

Although non-maleficence is a priority for the health professionals, a common theme in the media articles is that forced vaginal birth (denial of CSMR) may lead to trauma that remains with the woman for a long time, thus contradicting the do no harm-principle ^[S14, S12]^. At the same time, Sweden is one of the safest countries in the world for vaginal birth and has one of the lowest neonatal death rates, highlighting the non-maleficence aspect of vaginal birth. It is however argued, by one central member of the RVK-group, that forcing someone to do something they absolutely do not want (going through a vaginal birth) is “physical and psychological abuse” ^[S11]^. It is argued that the woman in those cases may have to spend the time after giving birth with processing a trauma instead of forming an attachment to the child, and in many cases the pregnancy is filled with confrontations with the healthcare services instead of preparations for becoming a parent ^[S12]^. In the media, there are many stories of trauma associated with *the process trying to get a CSMR* as well as trauma after *being forced to give birth vaginally*. Here is a woman describing the process: “*Fought for a whole autumn at [Hospital x] and became completely mentally broken. Was treated so condescendingly and superiorly*,* spitefully. Usually ended with me crying/shaking/hyperventilating/not getting air and then they wanted to end the conversation because I was ‘impossible to talk to’. Felt so much worse after my visits there*.” ^[S2]^ Another woman described that she was forced to give birth vaginally although she had explained that she was extra vulnerable to trauma due to a previous abusive relationship that caused PTSD and panic disorder: *“I brought this up to the Aurora midwife who waved it off with the rationale ‘the body is too busy giving birth’ to be traumatized.*” ^[S21]^ In the article it is also described that the woman was left alone during birth, without pain management, feeling fear of death and powerlessness, resulting in an emergency CS. However, there are also examples of health professionals saying that the healthcare services should not force phobic women to go through with a vaginal birth, but that the woman must be properly informed about the risks with a CS ^[S22]^. This illustrates that health professionals’ views are positioned along a scale in terms of risk tolerance.

### Beneficence: avoiding fear of birth and protecting bodily functions

In the media, it is argued that the desire to plan for a pain free and controlled birth is not strange (“pure self-preservation”) as the problems in birth clinics today are well-known, for example a lack of staff and many adverse outcomes such as severe tear injuries ^[S8]^. Predominantly, however, CSMR is portrayed in terms of fear of birth. According to presented statistics, every other woman is afraid or very afraid to give birth ^[S23]^. A midwife explains that almost all women have some kind of fear before giving birth but that for some women the fear takes over, and can become almost like a phobia. A woman expressed her fear like this: “*I have never been able to imagine giving birth normally. The whole body says no. The fear of it affects my everyday life so much that I get panic attacks when I think about it and severe anxiety. At night I wake up with nightmares. I have even had to pause my studies as it is always in the back of my mind.*” ^[S24]^ Among women requesting CS, the fear is often described as being about getting perineal or vaginal tears and that the own body will suffer, it is also about fear of not being listened to by the staff (it is seldom about the child) ^[S25]^. From this perspective, CSMR would be more in line with the principle of beneficence. An image that emerges in the media, largely conveyed by health professionals, is that fear of birth is linked to depression and mental ill health, and severe mental illness ^[S22, S26]^. A large group is also women who have suffered a previous traumatic or complicated birth. One view—expressed by a midwife—of why CSMR is not a good solution is that it does not “operate away mental ill health” ^[S27]^. Furthermore, vaginal birth is described as “natural”, “normal” and “healthy”. For example, a midwife also being a clinic manager says: “*Vaginal birth is the best medical approach for the mother as well as the child*” ^[S28]^, while another midwife says that “*Giving birth [vaginally] is the healthiest and most natural thing we do*” ^[S5]^.

A common opinion among health professionals is that a pregnant woman with a phobia should get therapeutic treatment ^[S23]^. In the media, health professionals describe that they put a lot of effort into supporting pregnant women so that they can give birth vaginally and feel safe. It is mentioned that they work with “de-dramatization of the birth process” ^[S13]^. The media picture is that many get good help from the Aurora-clinics and go through with a vaginal birth, while others perceive it as “a persuasion campaign” ^[S16,S29]^ and a “police interrogation” ^[S30]^. Another common approach is to offer pregnant women ‘birth contracts’, which means that the woman starts a vaginal birth, which can be converted into a CS if requested and assessed as medically safe. This is a way of offering women a sense of safety during birth, with conditions explicitly stated in writing and known to the caregivers. This is sometimes described as “a last resort” for women who wants a CS but are not granted one ^[S31]^, but may also be described as a compromise between different perspectives.

### Justice: regional variation and unclear costs

In the media, a number of arguments are made that reflect the perspective of third party-payers (the health system) and the society. A representative from the National Board of Health and Welfare explains that, from a societal perspective, it is not desirable with a larger share of CS ^[S15]^. This representative also maintains that although a review of CSMR recommendations is underway because of a variation in practice throughout the country, it is likely that they will remain the same. Many media articles illustrate that there is variation between the different birth clinics in how they interpret the recommendations and in their standpoint on when to perform CSMR ^[S13]^. One birth clinic is singled out as being extra reluctant towards CSMR. In addition, it is expressed that “the assessments are arbitrary” and that “being able to express your wishes and argue for your cause as a pregnant woman is crucial” ^[S12]^. This means that healthcare is not equal and that individual physicians’ attitudes become decisive. The media reports on different ways women try to find solutions and workarounds. “*Those interested know that women travel across the country*,* move addresses*,* send patient-referrals*,* call providers and advise each other on particular midwifes*,* or go abroad*” ^[S21]^. In one article, there is an example of a woman who was denied an elective CS in her home region and then decided to go to another one where her wish was granted, describing it as “God living in the city of Varberg” ^[S29]^.

There is also a debate about the needs-principle, and one opinion leader argues that if women have the right to choose CS (which is more expensive), it should be accompanied by the obligation to tell who will be down-prioritized ^[S14]^. Crowding-out effects are also mentioned as a drawback with more CSMR, and it is maintained that CSMR is not a private matter, but a public one as it is about the taxpayers’ money ^[S14]^. However, there is no consensus on how expensive CSMR is. Some debaters argue it is more expensive than vaginal birth, while members of RTK-group argue, for example, that “a prolonged vaginal birth that ends in acute CS is more expensive, also the aftercare due to injuries from vaginal deliveries” ^[S11]^. Rhetorically, the advocates for CSMR ask what the societal costs are from denying a woman a CS: *“How are usual complications from a vaginal birth valued? For example*,* pelvic floor problems*,* prolapse*,* incontinence*,* fecal leakage or not being able to enjoy sex as much anymore?”*^[S6]^.

## Discussion

The analysis shows that a struggle over how to relate to CSMR is taking place in Swedish media. It involves a confrontational approach and touches on all the ethical principles proposed by Beauchamp and Childress [29], which were used to analyze the media material. Many of the arguments put forward in the media are similar to those previously brought forward in an academic debate. The discussion shows that the principles are highly interlinked.

A core tension involves the principles of beneficence and non-maleficence, which are tightly intertwined and can be likened to two sides of the same coin. In the media’s portrayal of CSMR, it becomes apparent that health professionals focus more on ‘do no harm’ (non-maleficence) than on beneficence (even though vaginal birth is portrayed as being beneficial to the mother and child). Notably, health professionals and pregnant women who want a planned CS seem to have different opinions on what harm means. They seem to have different ideas of what the goal of the birth is and what potential adverse effects (risks) are acceptable/non-acceptable [24], and how these compare to each other. While the health professionals have predominantly a medical perspective on harm, pregnant women to a large degree also incorporate a psychological perspective and more holistic life perspective, for example avoiding a stressful pregnancy. It has previously been argued that the concept of risk is value-laden and that statistics on probabilities of different risks—such as duration of recovery, incontinence or future fertility—cannot tell us how different women value these outcomes [40]. It has therefore been suggested that the woman’s own risk tolerance must be part of the decision. In addition, the media’s portrayal of CSMR involves women’s contestation of the evidence health professionals refer to when explaining that vaginal births involve less risks than a CS (surgery). There are arguments that vaginal births are wrongfully compared to acute CS instead of planned CS, as well as concerns about the lack of evidence regarding the psychological effects of being forced to give birth vaginally against one’s will. This involves potential harm or trauma, and effects of reduced functioning, for example due to urinary and fecal incontinence, causing strain on relationships and work-life. Ultimately, the question is whether women should be autonomous to choose the risks they prefer (and avoid the risks associated with vaginal birth and ‘forced’ vaginal birth) as they are the ones who have to live with the consequences, or whether health professionals should have the right to make that risk assessment on their behalf. This protective approach can inadvertently undermine patient autonomy. Autonomy emphasizes the right of competent individuals to make informed decisions about their own healthcare. When a well-informed pregnant person requests a CSMR based on personal values, experiences, or fears, denying that request may be perceived as paternalistic, especially if the clinician’s decision is driven more by institutional norms or medicolegal concerns than by individualized risk assessment. This ethical tension highlights the need for shared decision-making, where clinicians provide comprehensive, evidence-based counseling and women are supported in making choices aligned with their values. Balancing non-maleficence with respect for autonomy requires nuanced judgment, cultural sensitivity, and a commitment to individualized care. However, even though non-maleficence for many health professionals outweigh patient autonomy [13], respect for autonomy is an important reason for some obstetricians to agree to elective CS [18].

Closely related to this is the question of the importance of information (and how to present and handle information). Burrow [41], for example, argues that “autonomy is more than a matter of having and pursuing options”. She argues that autonomy involves not only having sufficient information to make informed decisions, but also the ability to critically reflect on desires (such as those for CS birth) that may unconsciously reflect social preferences/pressure or norms. If not having the relevant information to adequately weigh risks and benefits or to appreciate how various options would affect one’s life, autonomy is limited. In line with previous research describing the child’s perspective as secondary, it is notable that partner pressure and the fetus’s perspective were largely absent from the debate, making it centered around the woman’s perspective. Balancing maternal autonomy with fetal well-being presents ethical challenges, particularly when maternal preferences may lead to avoidable neonatal risks. Additionally, tensions may arise if the partner disagrees with the decision for CSMR, underscoring the need for sensitive counseling and effective conflict resolution.

In the media portrayal, there is also a tension around whether pregnancy is to be seen as a specific condition to which specific rules should apply, or if pregnant women should be treated as any other patients seeking health care (compare with the current debate about normative and pragmatic reasons for regarding pregnancy as a disease or not [42, 43]). While many health professionals in our study argue that pregnant women should be treated as any other patients—which cannot choose surgery without medical indications—this position could also be used to defend the argument that pregnant women’s choice/autonomy should not be circumscribed, and that they should have the right to bodily integrity in the same way as other patients. Kingma [44], however, argues that autonomy and consent is even more important in maternity care as it involves interaction with body parts which are particularly sensitive and with a broader social significance. Autonomy and consent are more important than for less sensitive body parts such as knees or noses. In the media, the RVK-group uses the argument that pregnancy and birth is not like any other task—it is about bringing new lives into the world—and that it should therefore not be treated as a condition where only medical decisions are relevant. This is an argument that aligns with Robinson’s [45] suggestion that pregnancy gives women a temporary superior moral status because they are necessary for the continued survival of the human species. In line with this, Romanis ([24], p. 251) means that women should have the precedence to “decide what physical consequences of reproduction they are willing to assume”, while others (also in the Swedish media debate) argue that women must accept the premise that pregnancy ends with vaginal birth, with potentially unavoidable injuries, unless there are medical indications for CS. This is why Petersen [46], for example, argue that consent should not be necessary for vaginal birth; however maintaining that it is important to involve women in decisions on how to handle complications during birth. Vaginal birth being the ‘default form’ may be a reason why reproductive autonomy has hitherto focused mainly on whether and when to have sex, to become pregnant, and to use contraception, and whether or not to carry a pregnancy to term [47, 48]. Some however claim that it should also include “how the birth will happen” [49].

In the media’s portrayal, there is also a notable tension that involves the justice-principle. For those who argue against CSMR, two important aspects are the challenge to the needs-principle in Swedish healthcare and the questioning of how taxpayers’ money is allocated. In those arguments, the health system perspective (equal healthcare and priorities) is given priority over the individual’s needs or wishes. Autonomy is subordinate to the common good, and the argument that mode of birth is “a private matter” is rejected on the basis that it is paid for jointly through taxes, and that the resources may be better spent on other patients. This reasoning is reflected in the relatively weak patients’ rights in Sweden. Although the law (SFS 2014:821) emphasizes that patient autonomy must be respected, that the patient must be informed before giving consent, and “as far as possible” be consulted and involved in decisions and treatments, the interpretation of these principles appears particularly complex in the context of childbirth. For example, it is unclear whether consent is required for vaginal birth, what type of information must be provided, and what “as far as possible” means when it comes to being involved in choosing the mode of birth. This must also be balanced against law regulation (SFS 2010:659) establishing that healthcare professionals must perform their duties in accordance with science and proven experience. The latter supports the professionals’ view that CSMR is ultimately a professional decision, even if they strive for shared decision-making through information and support [38]. Transparent policies, equitable access to counseling, and shared decision-making can help mitigate the tensions between the justice principle and CSMR while respecting both individual rights and systemic fairness.

The arguments for and against CSMR emphasize the ever-present difficult balance between individual autonomy and the equitable allocation of limited healthcare resources. Ensuring equity often means placing limits on preference-based care to preserve access, fairness, and sustainability. One way to strive for balance would be to promote evidence-based guidelines that incorporate a broader range of perspectives than those currently considered, and to enhance the informed consent process. This is in line with the Birthrights movement in the UK where a strategic framework has been developed for the coming decade. In this framework it is stated that women and birthing people should have the right to make informed choices about their bodies, including mode of birth. This shows that this question is timely to write about, and a phenomenon debated worldwide [50]. Through the Swedish media portrayal, we identified a continued need to work on guidelines and practices for decision-making. Brione [51] emphasizes the importance of establishing ‘bilateral trust’, which includes, for example, critical reflection on patient’s values and a commitment to avoiding deception or coercion. The latter is according to the media portrayal experienced by many women with a wish for an elective CS. Brione also emphasizes that physicians should strive to understand what influences their own treatment preferences, be alert to the patient’s own risk tolerance, and help patients in identifying not only clinical choices but also moral issues they may face. In modern healthcare, individuals routinely undergo non-medically indicated procedures—such as cosmetic surgery, male circumcision, laparoscopic sterilization, and elective hysterectomy—even when less invasive or conservative treatments are available. Sometimes, these are publicly funded. These procedures are often justified on the basis of autonomy, psychological well-being, or personal values, and are typically performed with informed consent, without requiring extensive justification or resistance. CSMR, despite being similarly grounded in personal preference and bodily autonomy, is often met with greater scrutiny, resistance, or paternalistic attitudes. This may suggest an ambivalence in how autonomy is respected in pregnant versus non-pregnant individuals, and between women and men. A clearer adherence to these aspects in decision-making regarding CSMR could be a step in the right direction to reduce the current conflicts that are portrayed in the Swedish media. In addition, accountability must also be further clarified. If a vaginal birth is recommended but a CSMR is performed, will the obstetrician be held accountable for possible short term or long-term complications or should the pregnant woman take full responsibility? By contributing to the ongoing debate about CSMR in Sweden, we hope these results will support efforts to emphasize the importance of a national strategy with clearly defined criteria, leaving minimal room for arbitrariness and misunderstandings.

There are some limitations to this study. One is that we have only studied news media and specialist press, thus not capturing the debate taking place on social media. This would mean giving space to more voices, especially those that may not usually have such a prominent position in the public debate, and for other dimensions to emerge. Something that can be regarded a limitation, but also a strength, is the use of a framework for analysis. While contributing with an analytical focus, it may also limit the analysis to certain aspects, potentially leading to some aspects being missed, in this case other ethical principles than those proposed by Beauchamp and Childress [29].

## Conclusion

The Swedish media portrays a struggle over how to approach CSMR, involving a confrontational stance on all ethical principles proposed by Beauchamp and Childress. A key tension lies in how risks associated with vaginal birth and CSMR should be interpreted, weighed, and by whom. Another tension centers on whether a pregnant woman should be treated like any other patient who cannot choose treatments that are not medically indicated. A third tension involves that between the health system—and the principle to prioritize according to need-—and the individual’s preferences. Ultimately, this highlights the need for decision guidelines and practices that are more open to a woman’s own evaluation of different risks while also encouraging critical reflection on the underlying factors shaping treatment preferences for both women and health professionals.

## Supplementary Information

Supplementary Material 1.

Supplementary Material 2.

## Data Availability

Data material is available via the respective media sources.
