# Supplementary material for: The struggle over caesarean section on maternal request: an ethical principles approach to Swedish media portrayal
Source: Reprod Health. 2025 Jun 27;22:118. doi: 10.1186/s12978-025-02057-3 (PMC12203735; doi:10.1186/s12978-025-02057-3)
Supplement: Supplementary file 1 — Supplementary Material 1. [file 12978_2025_2057_MOESM1_ESM.pdf]

ADDITIONAL FILE 1

Table A1. Caesarean section (CS) rates by region and country[1]

Region/subregion/country	CS rate (%)	95% CI	Range (min-max, %)
**Africa (n=44)**	9.2	5.2–13.2	1.4–51.8
Northern Africa (n=5)	32.0	5.9–58.2	9.1–51.8
Sub-Saharan Africa (n=39)	5.0	3.5 – 6.6	1.4–50.7 8
**Asia (n=40)**	23.1	19.9–26.3	3.5–55.3
Central Asia (n=5)	12.5	6.5–18.4	5.3–18
Eastern Asia (n=5)	33.7	27.3–40.1	12.9–39.1
South-eastern Asia (n=8)	15.9	9.6–22.3	3.5–32.7
Southern Asia (n=7)	19.0	13.7–24.3	6.6–40
Western Asia (n=15)	31.7	22.7–40.6	4.8–55.3
**Europe (n=38)**	25.7	23.4–28.0	14.9–46.9
Eastern Europe (n=10)	25.0	18.7–31.3	17.9–46.9
Northern Europe (n=10)	25.3	21.5–29.1	15.9–32.6
Denmark	19.5		
Finland	16.4		
Iceland	18.3		
Norway	16.1		
**Sweden**	**17.4**	**Between 13–21% in the regions[2]**	
Southern Europe (n=11)	30.1	27.5–32.7	21.2–34.1
Western Europe (n=7)	24.2	18.3–30.2	14.9–32.7
**Americas (n=25)**	39.3	34.6 – 44.0	5.4–58.1
Latin America and the Caribbean (n=23)	42.8	37.6–48.0	5.4–58.1
Northern America (n=2)	31.6	20.5–42.8	28.8–31.9
**Oceania (n=7)**	21.4	6.6–36.2	3.0–34.6
Australia and New Zealand (n=2)	33.5	1.9 – 65.1	27.9–34.6
Melanesia, Micronesia, and Polynesia (n=5)	3.6	0.7–6.6	3.0–17.4
**World total (n=154)**	21.1	18.8–23.3	1.4–58.1
More developed countries (n=45)	27.2	25.2–29.2	14.9–55.3
Less developed countries (n=70)	24.2	20.9–27.5	2.4–58.1
Least developed countries (n=39)	8.2	5.2–11.2	1.4–32.7

1. Comparative statistics for caesarean section on maternal request are difficult to find. The majority of the data presented in this table comes from Betran AP, Ye J, Moller A-B, et al. Trends and projections of caesarean section rates: global and regional estimates. BMJ Global Health 2021;6:e005671. doi:10.1136/ bmjgh-2021-005671. Data for the Nordic countries are added from Global Health Observatory data repository, last updated 2018-04-09 (Births by caesarean section: Data by country). 2 SBU-report.
