# Supplementary material for: The struggle over caesarean section on maternal request: an ethical principles approach to Swedish media portrayal
Source: Reprod Health. 2025 Jun 27;22:118. doi: 10.1186/s12978-025-02057-3 (PMC12203735; doi:10.1186/s12978-025-02057-3)
Supplement: Supplementary file 2 — Supplementary Material 2. [file 12978_2025_2057_MOESM2_ESM.pdf]

ADDITIONAL FILE 2

Table A2. Media sources cited in the results section.

Ref	Type[1]	Article title in Swedish	Media source	Date	Author
S1	N	Socialstyrelsen ser över riktlinjerna för kejsarsnitt	SVT Nyheter	14 Oct 2019	Lena Pettersson
S2	D	Vi som vill ha kejsarsnitt blir förlöjligade och vilseledda	Göteborgs-Posten	7 Nov 2020	Rätten att välja kejsarsnitt
S3	O	Var förlossningen allt du hade hoppats på?	Svenska Dagbladet	2 Nov 2022	Jenny Björkman
S4	O	Barnafödande har blivit business på nätet	Göteborgs-Posten Plus	12 Apr 2021	Agnes Arpi
S5	N	Förlossningsrädsla får fler kvinnor att välja kejsarsnitt	Helagotland Premium	6 May 2019	Emelie Stenqvist
S6	N	"Det finns en bild av att kvinnan går miste om något vid kejsarsnitt"	Dagens Nyheter	3 Dec 2019	Hans Arbman, Thomas Lerner
S7	L	Murguz: "Rätt" till kejsarsnitt har inget att göra med kvinnans rätt till sin kropp	Borås Tidning	3 Jan 2020	Anela Murguz
S8	N	Väljer kejsarsnitt - på grund av rädsla Överläkaren: Vi människor behöver känna oss trygga	Aftonbladet	17 Feb 2020	Ulrika Lidbo
S9	D	Förlossningsvården ska vara till för patienten	Altinget	14 Sep 2021	Rätten att välja kejsarsnitt
S10	N	Vaginal förlossning eller kejsarsnitt - här är riskerna	Dagens Nyheter	2 Dec 2019	Hans Arbman, Thomas Lerner
S11	F	"Ett övergrepp att tvinga någon till en vaginal förlossning"	Dagens Nyheter	5 Dec 2019	Hans Arbman, Thomas Lerner
S12	D	Vi kräver en lex Rasha	Aftonbladet	30 Aug 2019	288 debaters
S13	N	Vårdens tävlingsmoment: Minska antalet kejsarsnitt	Göteborgs-Posten	25 Apr 2023	Magdalena Rosen, Agnes Arpi
S14	O	Kvinnokören får kejsarsnitt att framstå som en rättighet - det är det inte	Dagens Nyheter	14 Dec 2019	Hanne Kjöller
S15	N	"Inte önskvärt med fler kejsarsnitt"	Dagens Nyheter	5 Dec 2019	Hans Arbman, Thomas Lerner
S16	N	Kvinnor vittnar om motstånd till kejsarsnitt	Göteborgs-Posten	27 Oct 2020	Filip Mitrovic
S17	N	Det avgör om kvinnor får välja kejsarsnitt	Dagens Nyheter	17Nov 2023	Anna Bratt
S18	N	Det svenska kejsarsnittet - granskningen i korthet	Göteborgs-Posten Plus	4 May 2023	Agnes Arpi, Magdalena Rosen
S19	D	Kvinnor måste få välja hur de föder	Aftonbladet	15 Oct 2019	Jenny Wennberg
S20	N	Riktlinjer för kejsarsnitt på kvinnans önskemål saknas	Norran	28 Feb 2022	Ebba Andersson
S21	O	Säg som det är om kejsarsnitt på kvinnors egen begäran	Altinget	14 Feb 2022	Agnes Arpi
S22	N	"Förlossningsrädda är en känslig grupp"	Dagens Nyheter	13 Dec 2019	Hans Arbman, Thomas Lerner
S23	N	SKRÄCKEN: FÖDA BARN Aftonbladet/Demoskop: Hälften av kvinnorna lider av förlossningsrädsla	Aftonbladet	16 Feb 2020	Ulrika Lidbo

S24	N	Nekades planerat kejsarsnitt på grund av corona	Aftonbladet	15 Nov 2020	Malin Wigen
S25	N	"Att träffa samma barnmorska under graviditeten minskar oron"	Dagens Nyheter	10 Dec 2019	Hans Arbman, Thomas Lerner
S26	N	Så många kvinnor lider av förlossningsrädsla	Aftonbladet	14 Jan 2020	Ulrika Lidbo
S27	N	Ryktet: Hit ska du inte gå om du vill ha kejsarsnitt	Göteborgs-Posten Plus	26 Apr 2023	Agnes Arpi Hannah Zahr
S28	N	Fler föder med kejsarsnitt i länet: ”Vaginalt är bäst”	Folkbladet Västerbotten	27 Jan 2020	Anne Pettersson
S29	D	När gud bodde i Varberg sa en snittförlöst mamma om sin upplevelse	Hallands Nyheter Plus	6 Feb 2024	Per Strömberg
S30	F	Professor beklagar inslag av tävling i att hålla nere antalet kejsarsnitt	Svenska Dagbladet	23 Jun 2023	Karolina Bergström
S31	N	Förlossningskontrakt: Kvinnor med förlossningsrädsla tvingas börja föda vaginalt	Göteborgs-Posten Plus	26 Oct 2020	Filip Mitrovic

[1] N= News article/Feature article; O= Opinion piece; D= Debate article
